# *Adgrf5* contributes to patterning of the endothelial deep layer in retina

**DOI:** 10.1007/s10456-019-09674-0

**Published:** 2019-06-29

**Authors:** C. Niaudet, M. Petkova, B. Jung, S. Lu, B. Laviña, S. Offermanns, C. Brakebusch, C. Betsholtz

**Affiliations:** 1 Department of Immunology, Genetics and Pathology, Rudbeck Laboratory, Uppsala University, 75185 Uppsala, Sweden; 2 Department of Pharmacology, Max Planck Institute for Heart and Lung Research, Bad Nauheim, 61231 Germany; 3 Biotech Research and Innovation Centre, University of Copenhagen, Ole Maaløes Vej 5, 2200 Copenhagen, Denmark

**Keywords:** Retina, Adhesion receptor, *Adgrf5*

## Abstract

Neovascularization of the inner retinal space is a major cause of vision loss. In retinal angiomatous proliferation (RAP) syndrome, newly formed vessels originate from the retinal plexus and invade the inner retinal space. However, the molecular pathways preventing subretinal vascularization remain largely unknown. In most murine models of RAP, pathological neovascularization occurs concomitantly with the development of the retinal vasculature. Here, we demonstrate that disturbing the sequence of morphogenetic events that shape the three-layered retinal vascular network leads to subretinal vascularization. Sprouts emerging from the perivenous region after the first postnatal week extended toward the retinal space where they merged into the deep layer. The small GTPase Rac1 was required for the formation of these vascular extensions and the vascular inner plexus is formed coaxially to the overarching veins. The adhesion receptor *Adgrf5* was highly expressed in the endothelium of the central nervous system, where it regulates blood–brain barrier formation. The vascular superficial plexus of *Adgrf5* mutant mouse retinae exhibited an increased vascular density in the perivenous areas with increased projections toward the inner plexus where they subsequently created hyper-dense endothelial cells (EC) clusters. Disturbing the perivenous pool of EC thus significantly altered the inner plexus formation. These abnormalities culminated in transient vascular protrusions in the inner retinal space. Taken together, these results reveal a previously unobserved vascular morphogenetic defect in *Adgrf5* knockout mice, implicating a role for ADGRF5 in the initiation of subretinal vascularization. Our findings also illustrate how vein-derived EC shape the inner retinal layer formation and could control the appearance of angiomatous malformations.

**Electronic supplementary material:**

The online version of this article (10.1007/s10456-019-09674-0) contains supplementary material, which is available to authorized users.

## Introduction

The visual function of the eye is dependent on a strict organization of retinal tissue. The retina exhibits a multi-layered structure, in which the different cellular compartments and the vascular beds are organized to allow tissue oxygenation with minimal blockade of the passage of light. Thus, the neural retinal layer is ensheathed between two separate vascular beds. On the posterior side, the plexus of chorio-capillaries is the main oxygen supplier of the outer layers of the retina. On the inner (vitreal) side, the retinal vascular network develops into a stereotypical tri-layered architecture. The surface vascular plexus extends over the ganglionic cell layer (GCL) while two intra-retinal capillary networks radiate above the inner plexiform layer (IPL) and the outer plexiform layer (OPL), respectively [1, 2].

**Fig. 1:**
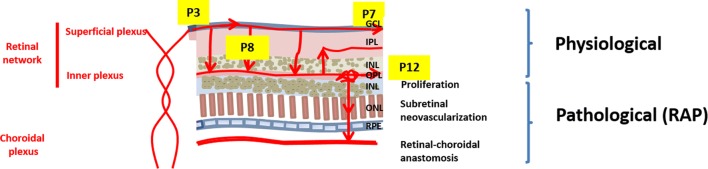
Retinal vascular network development. Scheme summarizing the development of the retinal network (*GCL* ganglional cell layer, *IPL* inner plexiform layer, *INL* inner nuclear layer, *OPL* outer plexiform layer, *ONL* outer nuclear layer, *RPE* retinal pigment epithelium). The lower scheme shows the progressive development of RAP lesions

The retinal vasculature first develops as a primary plexus sprouting from the optic nerve across the surface of the GCL. In the mouse, this process commences after birth. The superficial plexus expands and fully covers the retina by postnatal day 7 (P7). Numerous and detailed mechanisms that regulate this initial phase, including vascular endothelial growth factor (VEGF) and Notch signaling, have been well studied [3]. Soon thereafter, new sprouts expand into the retinal space to successively form the inner and the intermediary plexus, at P7 and P12, respectively (summarized in Fig. 1, “Physiological”) [2]. Major molecular cues guiding this second step have been proposed [4–6]. For example, VEGF secreted by the photoreceptor cells is the major driver of the deep layer formation. A progressive decrease in VEGF receptor 2 (VEGFR2) expressed by neurons sets the pace for blood vessel invasion, while local cues expressed by neighboring cell types constrain the expansion of the intermediary and the deep plexus [7].

Despite the extensive vasculature in the area, the photoreceptor layer is entirely devoid of any blood vessel under physiological circumstances. Currently, it remains unknown whether the avascular privilege in the photoreceptor layer has any biological function. A recent study has suggested that a hypoxic niche might be critical for proper neuroretina development by maintaining a pool of stem cells [7]. Moreover, vascular invasion of the neuroretinal space hampers optimal photo-transduction and can lead to irreversible blindness. Pathological invasion of the avascular inner retinal space may occur from either the choroidal or the retinal vascular network, leading to the conditions known as age-related macular degeneration (AMD) and retinal angiomatous proliferation (RAP), respectively. Both conditions cause severe impairment of the functionality of the retina and will ultimately damage vision [8, 9]. In patients affected by RAP, vascular sprouts first emerge from the deep vascular plexus above the OPL, bud inside the inner retinal space, and finally anastomose to the choroidal vasculature in the final stage of the pathogenic process (see Fig. 1, “Pathological”) [10, 11].

Unlike AMD, very few mutations have been linked with RAP in patients. Several loss-of-function mutant mice exhibit subretinal neovascularization, which evolves in the most severe cases into RAP. These studies suggest that the mechanisms leading to subretinal neovascularization might be closely associated with the ones governing normal deep retinal plexus development. Indeed, subretinal neovascularization often occurs in a narrow time window in mice, around P14 [12–16]. This coincides with a residual plasticity of retinal vascular development, where the vessels from the superficial plexus layer begin to project down to generate the nascent deeper plexus layer. The descending vessels are funneled through the action of local cues such as semaphorins and disturbing these cues results in hypovascularization [4–6]. Conversely, altering the distribution of VEGF leads to subretinal neovascularization, which evolves into a full RAP syndrome. Physiologically, a rigorous protection against VEGF is maintained by the steady production of sFlt1 from the neuroretina [17].

Control of cellular polarity is essential to retinal morphogenesis [18]. Adhesion G protein-coupled receptors (aGPCRs) have been shown to control different types of polarity-related functions [19] such as oriented cell division [20], planar cell polarity [21, 22], and cell migration [23]. As such, aGPCRs are involved in regulating numerous developmental processes [24]. aGPCRs exert a part of their biological action through a long extracellular *N*-terminus bearing adhesive domains. These domains allow the receptors to interact with membrane-tethered partners or molecules embedded within the extracellular matrix, a feature that renders them unique within the GPCR superfamily [25, 26]. *Adgrf5* is one of the 5 aGPCRs highly expressed in the central nervous system (CNS) endothelium [27]. Suppression of *Adgrf5* in the endothelium impairs the function of the blood–brain barrier (BBB), but no morphogenetic defect in the CNS capillaries has been documented thus far [28]. In parallel, *Adgrf5* knockout (KO) mice exhibit overproduction of surfactant in the lungs, linked with the expression of *Adgrf5* in the type II alveolar epithelial cells [29–31].

In the present study, we assessed the impact of *Adgrf5* deficiency on the formation of the inner vascular layer in the retina. Our results demonstrate that subretinal vascularization is intimately associated with the sequence of morphogenetic events that shape the superficial plexus. The vascular inner plexus developed coaxially to the overarching veins. In *Adgrf5* KO mice, the vascular superficial plexus exhibits increased vascular density in the perivenous areas, creating increased EC projections toward the inner plexus where they form hyper-dense cell clusters. These abnormalities culminate in the formation of transient vascular protrusions in the inner retinal space. Taken together, these results illustrate how vein-derived EC contribute to the inner retinal layer formation and control the emergence of angiomatous proliferation. Our data also show the first vascular morphogenetic defect observed in *Adgrf5* KO mice, and suggest that ADGRF5 deficiency mimics the initiation step of RAP.

## Materials and methods

### Ethics statement

Animal housing, as well as the experiments performed, was in accordance with Swedish legislation and was approved by the local animal ethics committees prior to experimentation. The protocols included in this study were approved by the Uppsala Committee (permit number C224/12). Animal suffering was minimized, and all surgical procedures were performed under anesthesia (Ketamine (75 mg/kg) and Dexdomitor (0.5 mg/kg)).

### Mouse models

#### *Adgrf5* knockout

*Adgrf5* KO mice have been described previously [28]. Briefly, the *Gpr116*^−/−^ Velocigene mouse line was generated by Regeneron using a VelociGene approach [32] to delete exon 3 to exon 21. Heterozygous mice were backcrossed to C57BL6 mice, for at least six generations. Knockout and heterozygous pups were identified through genotyping PCR using the following primers: forward: 5′-GGGTAACGTGCTCTCTCTGC-3′, reverse wildtype: 5′-TGAACTCCTGGATACTAGCC-3′, or reverse knockout: 5′-TCATTCTCAGTATTGTTTTGCC-3′). The predicted amplicon sizes are 325 base pairs (bp) for the *Adgrf5* wild-type (WT) band, and 401 bp for the knockout allele.

#### *Claudin5*-GFP

The transgenic mouse *Cldn5*-GFP, expressing eGFP under the control of the *Claudin5* promoter, was generated using Bacterial artificial chromosome (BAC) technology. Complete characterization of this endothelial transgenic line will be presented in an independent manuscript. The line was kept in a C57/Bl6 background (*Cldn5*-GFP) or bred with the *Adgrf5* KO (*Adgrf5* KO × *Cldn5*-GFP).

#### *Rac1* ECKO

Endothelium-specific tamoxifen-inducible Rac1 knockout mice (referred to as “*Rac1* ECKO“) were obtained by crossing *Rac1*^flox/flox^ mice (floxed exon 3) [33] with the endothelial-specific *Cdh5*(PAC)-CreERT2 mice [34]. To evaluate the recombination efficiency, *Rac1* ECKO mice were bred into the *Rosa26*-EYFP reporter mouse [35]. Postnatal gene deletion was induced through maternal milk by tamoxifen (Sigma-Aldrich, #T5648) gavage of the dam from P1 to P3. Mice were sacrificed at P8.

*Rac1* ECKO pups were identified through genotyping using the following primers: forward 5′-TCACTGCTTGTCTCATGC A-3′, reverse 5′-AACGTATGGTTTCATTAAACTGA-3′. The predicted amplicon size is 318 bp for the floxed *Rac1* allele and 236 bp for the WT allele. Cre-positive pups were identified through the following primers: forward: 5′-GATATC TCACGTACTGACGG-3′, reverse 5′-TGACCAGAGTCATCCTTAGC-3′. The predicted amplicon size is 300 bp for the Cre-positive band.

#### *Adgrf5* ECKO

As described previously, *Adgrf5* floxed exon 17 mice, generated by Novartis [29], were crossed to mice expressing a constitutive *Tie2*-Cre allele [36]. Offspring was intercrossed to generate mice homozygous for the floxed *Gpr116* allele, and carrying one copy of *Tie2*-Cre. The male offspring was crossed *to ROSA26*mT/mG females (Jackson Laboratory, Stock No. 007576) to allow tracking of the recombined endothelial cells. This line, *Flox Gpr116* × *Tie2*-Cre × *ROSA26*mT/mG, is referred to as “*Gpr116* ECKO.”

#### *Adrgf5* Δ17

*Adgrf5* Δ17 mice, harboring a deletion into the *Adgrf5* exon 17, which encodes for the transmembrane domains, were generated by germ line deletion. Briefly, a male carrying the floxed exon 17 was crossed to a *Tie2*-Cre-positive female mouse, since the activation of Cre recombinase by the *Tie2* promoter occurs during oocytogenesis. Thus, the floxed exon will be removed in the germ line, ultimately resulting in global deletion of the gene [37]. The efficiency of the LoxP/Cre recombination in the offspring was examined by PCR to select mutants that have lost exon 17 using the following primers: forward 5′- TGTTAACCTCTGGCCTCTGC-3′, reverse 5′- TGTTAACCTCTGGCCTCTGC-3′ [29]. The predicted amplicon size is 490 bp.

#### *Gpr116-m*Cherry

Transgenic mice expressing mCherry under the control of the *Gpr116* promoter (*Gpr116*-mCherry) were generated as described before using the BAC clone RP24-510M8 (CHORI, CA, USA) from mouse chromosome 17 containing the *Gpr116* gene [38]. For genotyping by PCR the following primers were used: forward: 5′-CTTCATCATGTCCACAGAACC-3′; reverse: 5′-AGGATGTCCCAGGCGAAGG-3′; PCR products of 502 bp indicate the transgenic allele.

#### *Adgrf5* ECGOF

The gain-of-function Gt(*Rosa26*-*CAG*-*Adgrf5*) mouse model expresses a conditional knock-in allele of *Adgrf5* under the control of a CAG promoter (Taconic).

To overexpress *Adgrf5* in the endothelium, a Gt(*Rosa26*-*CAG*-*Adgrf5*) mouse was crossed to the *Tie2*-Cre line to yield “*Adgrf5* endothelial cell gain-of-function” (*Adgrf5* ECGOF) mice. The transgene was detected using the following primers: forward 5′-TGGCAGGCTTGAGATCTGG-3′, reverse 5′-CCCAAGGCACACAAAAAACC-3′, control forward primer 5′-GGGGCAATCAATTGAGGG-3′, control reverse primer 5′-CAACCTCTGCTTGGTTCTGG-3′. PCR products of 492 bp indicate the presence of the conditional knock-in allele and the 333 bp control band the presence of a WT allele.

### Tracer injection

To assess cerebral vascular leakage, 1 kDa Alexa Fluor 555-conjugated cadaverine (Life Technologies) was injected intravenously into the tail vein in adult mice (2 months). After 2 hours (h), the anesthetized animals were perfused with Hanks’ balanced salt solution (HBSS) for 5 minutes (min), brains were harvested and homogenized in 1% Triton X-100 in PBS (pH 7.2). Brain lysates were centrifuged at 13,000 rpm for 20 min at 4 °C and the relative fluorescence of the supernatant was measured on a Synergy HT271167 plate reader (excitation/emission 540/590 nm). After HBSS perfusion, tracer extravasation into brain parenchyma was visualized with a Leica stereomicroscope [39].

### Organ processing

Mice were anesthetized, and intracardiac perfusion of HBSS was performed.

For retinal whole mount staining, retinae were fixed in 4% PFA in PBS for 1 h at room temperature (RT), dissected, permeabilized at 4 °C overnight, rinsed in PBS, washed twice in PBlec (1% Triton-X100, 0.1 mM CaCl_2_, 0.1 mM MgCl_2_, 0.1 mM MnCl_2_ in PBS, pH 6.8), and incubated for 48 h at 4 °C with Alexa Fluor® 647-conjugated isolectin B4 (Invitrogen), as well as primary antibodies (CD31(Abcam), Erg (Abcam), ASMA (Sigma-Aldrich), all 1:500). After three washes in PBS, retinae were incubated for 2 h at RT with secondary antibodies, 1:400 diluted in PBS, 0.5% bovine serum albumin (BSA), and 0.25% Triton X-100. Retinas were washed and flat-mounted in ProLong Gold mounting medium (Life Technologies).

For retinal histology, eyeballs were fixed in 4% PFA in PBS for 24 h at 4 °C. They were subsequently embedded in paraffin and 5 μm sections were prepared using a HM355S microtome (Thermo Fisher Scientific). After deparaffinization, sections were stained with eosine and with Mayer’s hematoxylin. Finally, slides were dehydrated and mounted in Neo-Mount (Merck). Alternatively, dehydrated slides were post-fixed in 4% PFA for 1 h, permeabilized for 2 h, and incubated overnight with primary antibodies (collagen IV (AbD Serotec Bio-Rad), NeuN (Merck), GFAP (Zymed labs), TER-119 (BioLegend), all 1:100), and Alexa Fluor® 647-conjugated isolectin B4. After three washes in PBS, the sections were incubated for 2 h at RT with secondary antibodies and Hoechst, and mounted in ProLong Gold mounting medium (Life Technologies).

For lung histology, the trachea was exposed and a 20-gauge blunt needle was inserted, perfused with a 4% PFA solution to inflate the lungs, which were post-fixed overnight in 4% PFA solution. For paraffin sections, lungs were embedded in paraffin and 8-µm-thick sections were prepared using a HM355S microtome. After deparaffinization, sections were incubated 10 min in Mayer’s hematoxylin, followed by eosin. Finally, slides were dehydrated and mounted in Neo-Mount (Merck).

### Confocal microscopy and 3D retinal reconstruction

All confocal images were acquired with a Leica TCS SP8 confocal equipped with a tunable white light laser, and processed using Photoshop (Adobe). Images were assembled using the LASX software (Leica). 3D structures were constructed using the “Blend” volume representation of the LASX software. The depth color coding was used to represent better the depth of the layers, coloring in warm colors the superficial plexus, and colder colors the deeper ones.

### Quantifications

For quantifications, images were analyzed using ImageJ software (NIH).

For samples from *Adgrf5*-mCherry mice, the fluorescence intensity was calculated for each vascular bed proximal to an artery or a vein, respectively (within a 100 µm distance), and averaged for three retinae per age.

For vessels extending from the superficial to the inner plexus, vessels were quantified on whole retinas 3D pictures along each full artery or vein, using the first capillary bifurcation as the lateral limit. Three retinae were used per genotype.

For inner plexus densities, the vascular density plugin was used on 500 µm^2^ areas inside the inner plexus of three retinae per genotype. To further characterize the capillary organization, the Analyze particles plugin was used and the resulting perimeters of the vascular structures distributed by size (0 to 10 µm, 10 to 100 µm, 100 to 1000 µm, over 1000 µm).

### mRNA extraction and quantification

Whole snap-frozen organs were homogenized using the FastPrep-24 (Bertin) machine. Total RNA was extracted with RNeasy Mini Кit (QIAGEN) following the manufacturer’s protocol. The RNA quantity was measured on a Synergy HT Microplate machine (BioTek). The RNA quality was determined by the ratio of absorption A260/A280, and was considered as acceptable in the range of 1.9–2.1.

### Quantitative RT-PCR on organs and EC

cDNA was synthesized from 0.5 μg total RNA using the iScript cDNA Synthesis Kit (Bio-Rad). Real-Time quantitative PCR (qRT-PCR) was performed using TaqMan® Gene Expression Master Mix (Life technologies) on a CFX96 Touch™ Real-Time PCR Detection System (Bio-Rad). The expression level was normalized to Hprt as a housekeeping gene.

The following probes were used: mouse *Adgrf5* (Assay: Mm01269028_m1, FAM, spanning exon 17–18, Life Technologies), *Pecam*-1 (Assay: Mm01242576_m1, FAM, Life Technologies), *Hprt* (Assay: Mm03024075_m1, FAM, Life Technologies).

### Statistical analysis

Data are expressed as mean ± SD. In datasets containing two distinct groups, statistical comparisons were performed with the Student’s *t* test, and *p* < 0.05 was considered statistically significant. In dataset containing three distinct groups, statistical comparisons among groups were performed using one-way ANOVA followed by Tukey’s post hoc test and *p* < 0.05 was considered statistically significant. On the figures, the error bars represent SD, and *p* < 0.05 is represented as *p < 0.005 as **p < 0.0005 as ***p < 0.00005 as **** and “ns” stands for “no significant difference.”

## Results

### The inner plexus develops coaxially to the overarching veins

**Fig. 2:**
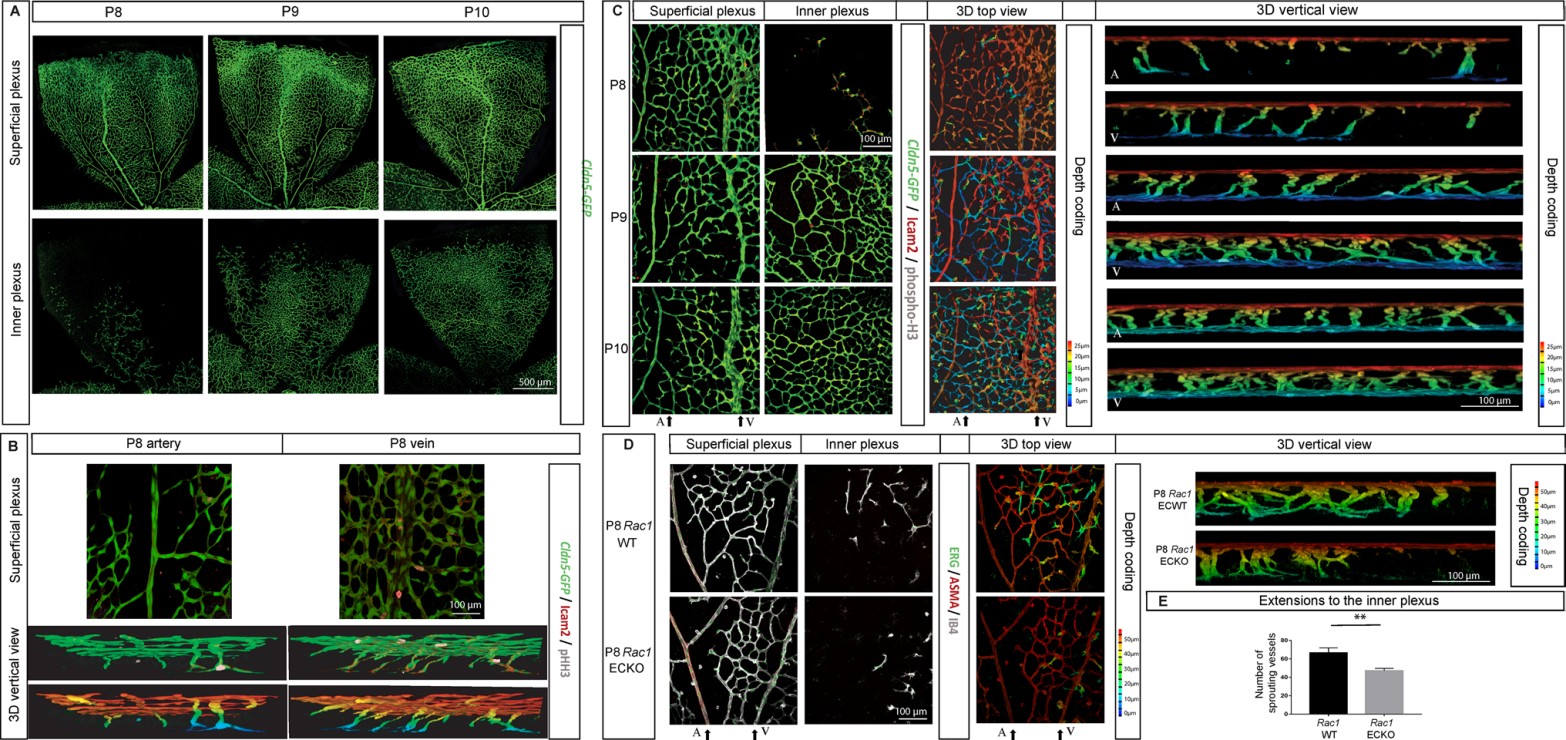
Retinal vascular inner plexus develops coaxially to the overarching veins. **a** Morphology of the superficial (upper) and inner (lower) vascular plexus from *Cldn5*-GFP transgenic reporter mouse retina at P8, P9, and P10. The vascular network is genetically labeled (GFP, shown in green). “A” and “V” indicate the position of an artery and a vein, respectively. **b** Visualization of the superficial vascular layer around an artery (left) and a vein (right) of *Cldn5*-GFP mouse retina at P8. The EC projections toward the inner plexus specific to each area were reconstructed (3D vertical view). Retinal EC were genetically labeled by *Cldn5*-GFP (green) and further stained for ICAM2 (red) and phospho-histone H3 (pHH3, gray) to visualize vascular lumen and proliferation, respectively. Depth coding of the same area is shown. **c** Superficial (left), inner (middle left) vascular plexus, and 3D overlay view from top (middle right) of *Cldn5*-GFP mouse retina at P8, P9, and P10. 3D vertical view below the artery or the vein is shown (right). **d** Superficial (left), inner (middle left) vascular plexus, and 3D top view (middle right) of WT and *Rac1* ECKO retina. A vertical view below the vein (V) is shown (right). Artery (A) and vein (V) are indicated. **e** Quantification of the EC projections to the inner plexus observed below the vein in *Rac1* ECKO and WT mouse retina at P8

Using a transgenic reporter mouse line that labels EC, *Cldn5*-*GFP*, we first studied the physiological progression of retinal vascularization in otherwise WT mice. We observed that the inner plexus develops following a stereotypical pattern during mouse retinal development. In a C57/Bl6 background, the first sprouts that emerge from the superficial plexus started to form the inner plexus around P8, which reached the retinal periphery at P9 and covered the entire retinal surface at P10 (Fig. 2a, “Superficial plexus”). Furthermore, we found that the inner plexus emerged below the veins and surrounding capillaries of the superficial plexus at the initial stage (P8), and these initial vascular patches became denser at later stages (P9 and P10, Fig. 2a, “Inner plexus”), while the rest of the inner plexus kept extending radially.

To understand this coaxial progression, we tracked the vascular sprouts diving from the superficial plexus that form the inner vascular network at P8. Numerous vertical sprouts were detected below the perivenous area, in contrast to the arterial region (Fig. 2b, “3D vertical view”). The perivenous sprouts showed a highly developed and complex pattern, as revealed by immunofluorescence staining with the luminal marker, ICAM2 (Fig. 2b, “3D vertical view”). This coincided with a dense capillary network around the veins in the superficial plexus, as compared to the sparse capillary network surrounding the artery (Fig. 2b, “Superficial plexus”).

Once initiated at P8, the numerous vertical vascular projections and the resulting patches of high density in the inner plexus below the veins (Fig. 2c, “3D vertical view” and “Inner plexus”) were still observed while the inner plexus developed at P9 and P10 (Fig. 2c, Inner plexus). Meanwhile, the perivenous capillary network in the superficial plexus was progressively normalized (Fig. 2c, “Superficial plexus”).

We tested whether active EC migration is required to prolong the high EC density in the perivenous area. Rac1 is a Rho GTPase critical for cell migration [40]. Therefore *Rac1* gene was abrogated postnatally, using conditional endothelial-specific *Rac1* knockout mice (*Rac1* ECKO). In mutants, the number of sprouts derived from the perivenous area that reached the inner plexus was reduced at P8 (Fig. 2d, “Inner plexus” and “3D vertical view,” quantified in Fig. 2e). However, the overarching superficial network exhibited an unaltered density (Fig. 2d, “Superficial plexus”). These results suggest that the inner plexus formation requires EC endowed with intact Rac1 activity in contrast to the formation of the superficial plexus, which develops independently of Rac1.

### *Adgrf5* is exclusively endothelial in the retina

**Fig. 3:**
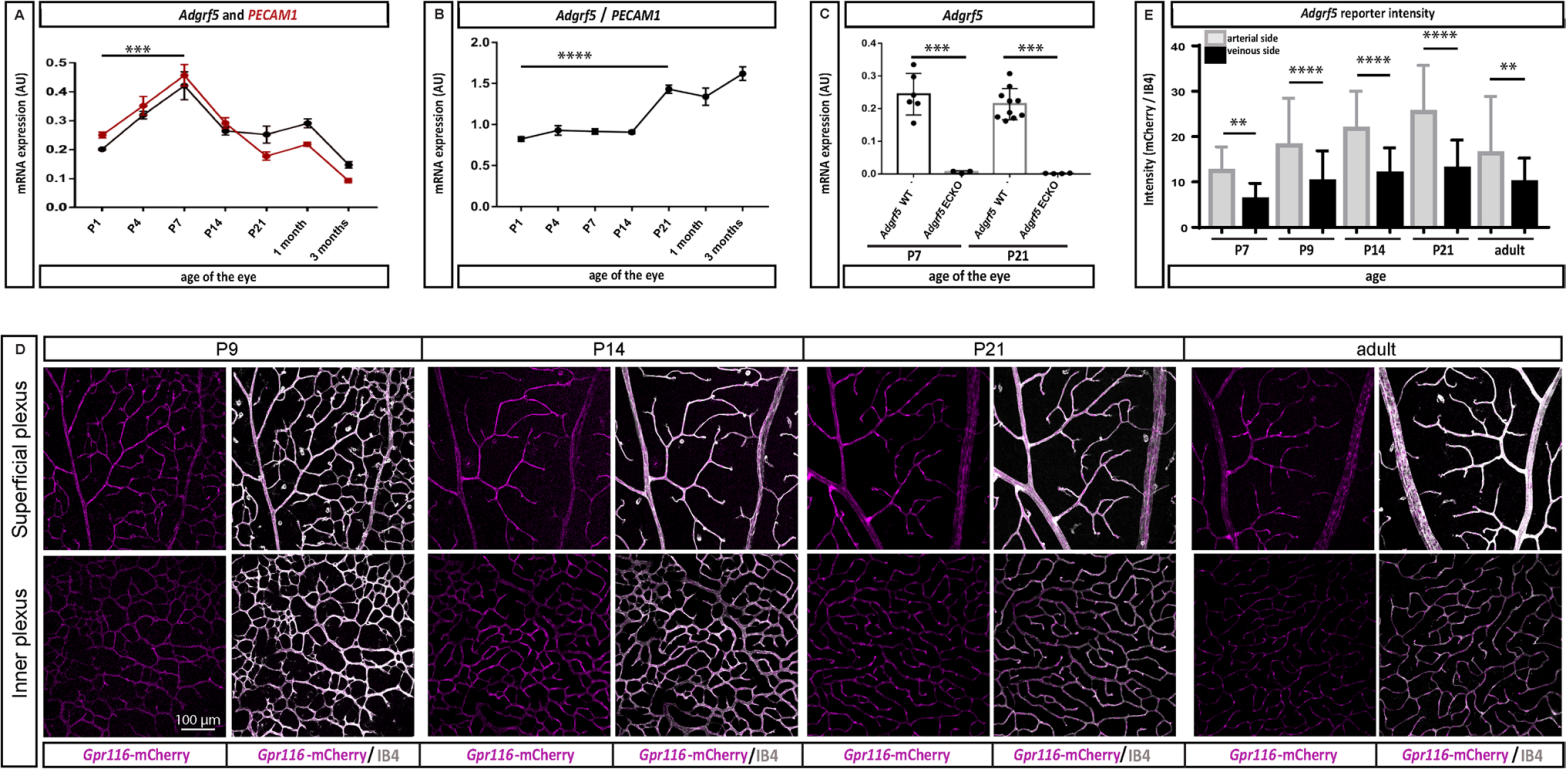
*Adgrf5* is exclusively endothelial in postnatal retina. **a** mRNA expression levels of *Adgrf5* (black line) and *Pecam*-1 (red line) in mouse retina. The whole retinal RNA from C57BL6 mice was used to determine *Adgrf5* and *Pecam*-*1* transcripts by qRT-PCR at P1 (*n* = 6 mice), P4 (*n* = 4), P7 (*n* = 3), P14 (*n* = 5), P21 (*n* = 5), 1 month (*n* = 3), and 3 months (*n* = 4). The expression of each gene was normalized by *Hprt*. Error bars represent ± SD. **b** Relative mRNA expression level of *Adgrf5* normalized by *Pecam*-*1* in C57/Bl6 retinae. The same number of animals mentioned in Fig. 2a was used. **c***Adgrf5* mRNA expression level over the course of the eye development. *Adgrf5* WT and *Adgrf5* ECKO mouse retinal RNA was prepared at the time indicated and *Adgrf5* transcript was assessed by qRT-PCR (*Adgrf5* WT (*n* = 6) and *Adgrf5* ECKO (*n* = 3) at P7 and *Adgrf5* WT (*n* = 10) and *Adgrf5* ECKO (*n* = 4) at P21). Results are normalized by *Hprt* expression. **d** Fluorescent images of ADGRF5 expression in-between an artery and a vein in superficial (upper) and inner (lower) vascular layer. *Gpr116*-mCherry reporter mouse retinae were collected at the time indicated, and stained with IB4 (gray) to visualize the endothelium. mCherry indicates *Adgrf5* expression (magenta). **e** Quantification of the fluorescence intensity in *Gpr116*-mCherry reporter mouse retinae, normalized over isolectin (lB4) fluorescence

We and others have shown that *Adgrf5* is expressed in the endothelium of all vascular beds of major organs in mice, though non-EC cell types might also express it in some tissues [28, 31]. In the specific case of the retinal vasculature, we first measured the mRNA expression of *Adgrf5* at various stages of development by real-time PCR. In C57/Bl6 animals, *Adgrf5* and the endothelial marker *Pecam*-*1* revealed a similar expression pattern with a nearly twofold increase over the first week of postnatal development followed by a threefold, and a fivefold decrease up to the third month of development for *Adgrf5* (0.15 ± 0.02, *p* ≤ 0.0001) and *Pecam*-*1* (0.09 ± 0.02, *p* ≤ 0.0001), respectively (Fig. 3a). These results indicate that the mRNA level of *Adgrf5* is strongly correlated with the expression of the endothelial marker *Pecam*-*1*. Overall, the ratio of *Adgrf5* to *Pecam*-*1* transcripts showed a steady profile from P1 to P14 followed by a 1.7-fold increase (0.9 ± 0.4 to 1.62 ± 0.17, *p* = 0.0002) from P7 to P14, and plateaued again during adulthood (Fig. 3b).

To determine whether the *Adgrf5* gene is exclusively expressed by EC in the postnatal retina, we generated a constitutive, endothelial-specific deletion mouse model of ADGRF5 (“*Adgrf5* ECKO”), by crossing a mouse carrying a floxed *Adgrf5* exon 17 to a *Tie2*-Cre mouse line [41]. Our real-time PCR data revealed that the *Adgrf5* mRNA level in *Adgrf5* ECKO mouse retina (0.006 ± 0.004) was reduced to 2.4% of the WT littermates (0.244 ± 0.06, *p* ≤ 0.0001) at P7. At P21, *Adgrf5* ECKO mice showed 0.5% of the *Adgrf5* transcript levels compared to littermate control retina (0.001 ± 0.0001 for *Adgrf5* ECKO, 0.196 ± 0.04 for WT, *p* ≤ 0.0001) (Fig. 3c). These results suggest that, upon genetic ablation of *Adgrf5* in the endothelium, the expression level of *Adgrf5* in the retina is completely abrogated, hence excluding expression in other cell types than EC.

Next, we employed a complementary approach to confirm the endothelium-specific expression of *Adgrf5*, and analyzed the retinal vasculature of the *Gpr116(Adgrf5)*-mCherry mice, which express mCherry under the control of the *Adgrf5* promoter and its enhancer elements [38]. Expression of *Adgrf5* co-localized with the isolectin B4 (IB4)-positive endothelium in the superficial plexus at P9, indicating EC-specific ADGRF5 expression in the retinal vasculature. Starting at P14, *Adgrf5* expression in the inner plexus was also observed. The strong expression pattern of *Adgrf5* in the superficial plexus was maintained over the course of the retinal vasculature development (Fig. 3d). Specifically, the highest reporter activity was observed on the arteriole side and branching points within the superficial plexus (Fig. 3e). Reporter expression was further detected in the horizontal cells below the outer nuclear layer in 3-month-old animals (data not shown).

### *Adgrf5* deficiency leads to increased vascular density around the veins in retina

**Fig. 4:**
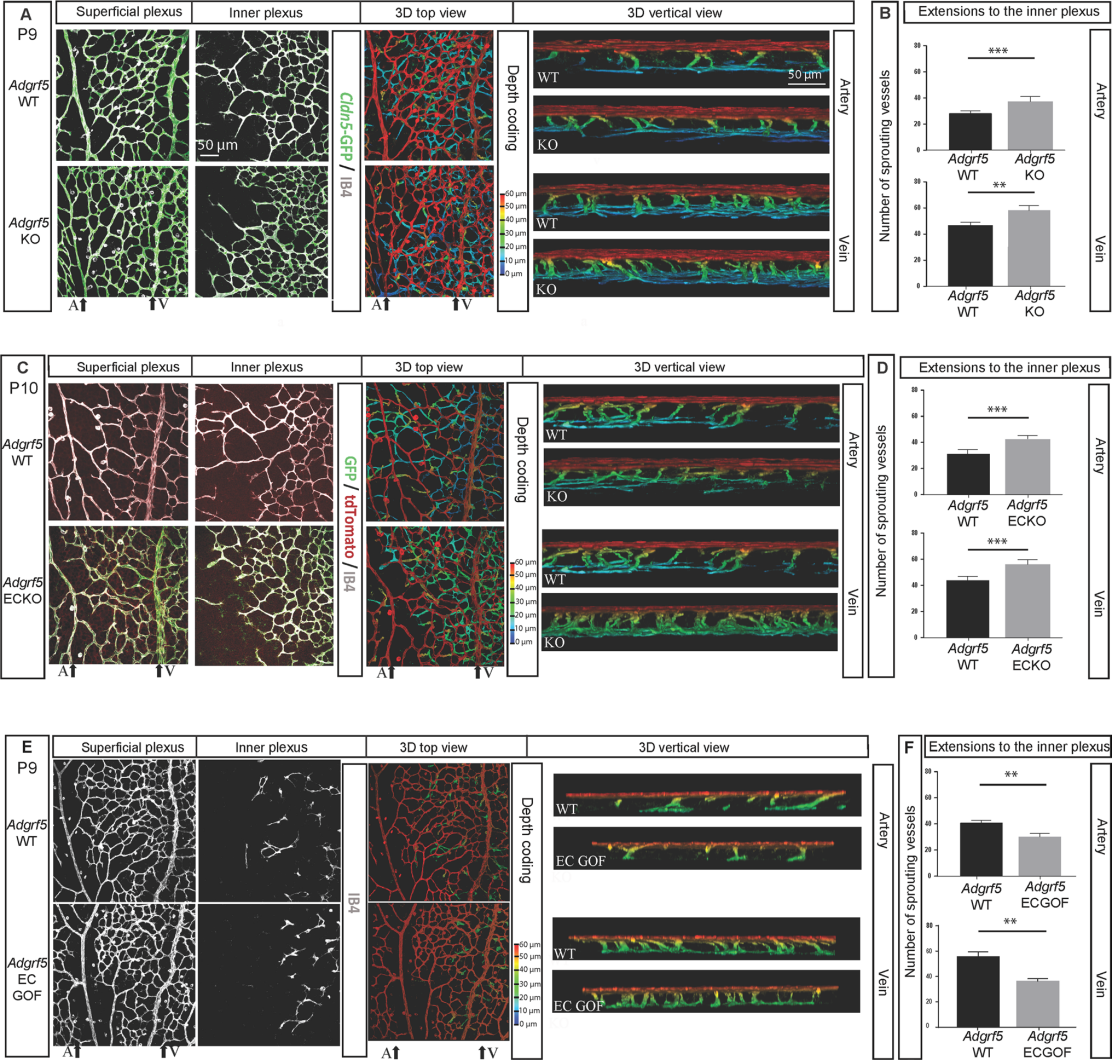
*Adgrf5* deficiency leads to perivenous abnormalities. **a** Involvement of *Adgrf5* in the inner plexus formation. Superficial (left) and inner (middle left) vascular plexus in-between an artery and a vein from WT (upper) and *Adgrf5* KO (lower) retina at P9 are shown. The endothelium is visualized by *Cldn5*-GFP reporter (green) and IB4 staining (gray). Complete 3D reconstruction of the same area is represented in depth coding with a top view (middle), and a vertical view below the vein (V) and an artery (A) is indicated (right panel). **b** Quantification of the number of extensions from the superficial to the inner vascular layer in WT and *Adgrf5* KO under the arteries (top) and under the veins (bottom). **c** Role of EC-specific *Adgrf5* in the inner plexus development. Superficial (left) and inner (middle left) vascular layer in-between an artery and a vein in the WT (upper) and *Adgrf5* ECKO (lower) retina at P10 are shown. The endothelium is visualized by IB4 staining (gray). *Adgrf5*-depleted EC are marked by GFP (green). Complete 3D reconstruction of the area from WT and *Adgrf5* ECKO is represented in depth coding with a top view (middle), and a vertical view below the vein (V) and an artery (A) is indicated (right panel). **d** Quantification of the EC protrusions from the superficial to the inner vascular layer in WT and *Adgrf5* ECKO under the arteries (top) and under the veins (bottom). **e** Role of EC-specific *Adgrf5* in the inner plexus development. Superficial (left) and inner (middle left) vascular layer in-between an artery and a vein in the WT (upper) and *Adgrf5* ECGOF (lower) retina at P9 are shown. The endothelium is visualized by IB4 staining (gray). Complete 3D reconstruction of the area from WT and *Adgrf5* ECGOF is represented in depth coding with a top view (middle), and a vertical view below the vein (V) and an artery (A) is indicated (right panel). **f** Quantification of the EC protrusions from the superficial to the inner vascular layer in WT and *Adgrf5* ECGOF under the arteries (top) and under the veins (bottom)

The expression pattern of *Adgrf5* in the superficial plexus prompted us to re-examine retinal vasculature in *Adgrf5*-deficient mice. We previously showed that *Adgrf5* KO retinal vasculature did not exhibit any obvious growth defect at P4. However, P9 retina from *Adgrf5* KO animals revealed a denser retinal capillary network around the vein as well as an increased number of inner sprouts detected below the perivenous area, and a more developed inner plexus (Fig. 4a, “Superficial plexus” and “Inner plexus”). These results suggest that the increased inner sprouts observed in *Adgrf5* KO mice (Fig. 4b) might have accelerated the formation of a denser inner network. Additionally, *Adgrf5* ECKO mice showed a similar pattern as *Adgrf5* KO, specifically an increase of the vascular density around veins and arteries in the superficial plexus (Fig. 4c, “Superficial plexus”) and a higher number of inner sprouts (Fig. 4d). We found that, once the sprouts formed the inner plexus, it became denser and more complex in mutants than in littermate WT (Fig. 4c, “Inner plexus”). These data indicate that endothelial ADGRF5 is involved in the regulation of the perivenous capillary density, which subsequently influences inner plexus formation.

As genetic ablation of *Adgrf5* in mice causes a denser vasculature, both in the superficial and in the inner plexus, we addressed the effect of *Adgrf5* overexpression. To this end, we generated a gain-of-function *Adgrf5* mouse model. Specifically, *Adgrf5* gene was overexpressed in the endothelium by crossing the Gt(*Rosa26*-*CAG*-*Adgrf5*) mouse to a *Tie2*-Cre line. These mice, named *Adgrf5* endothelial cell gain-of-function (*Adgrf5* ECGOF), showed a sparse perivenous network in the retinal vasculature at P9 (Fig. 4e). Moreover, a low number of endothelial sprouts protruded through the inner nuclear layer (INL) (Fig. 4f), and the inner plexus was sparse compared to WT littermates. Taken together, these data suggest that the level of endothelial ADGFR5 is critical for regulating the perivenous vascular density in the superficial plexus, thus tightly controlling the inner plexus density.

### Loss of ADGRF5 triggers subretinal neovascularization

**Fig. 5:**
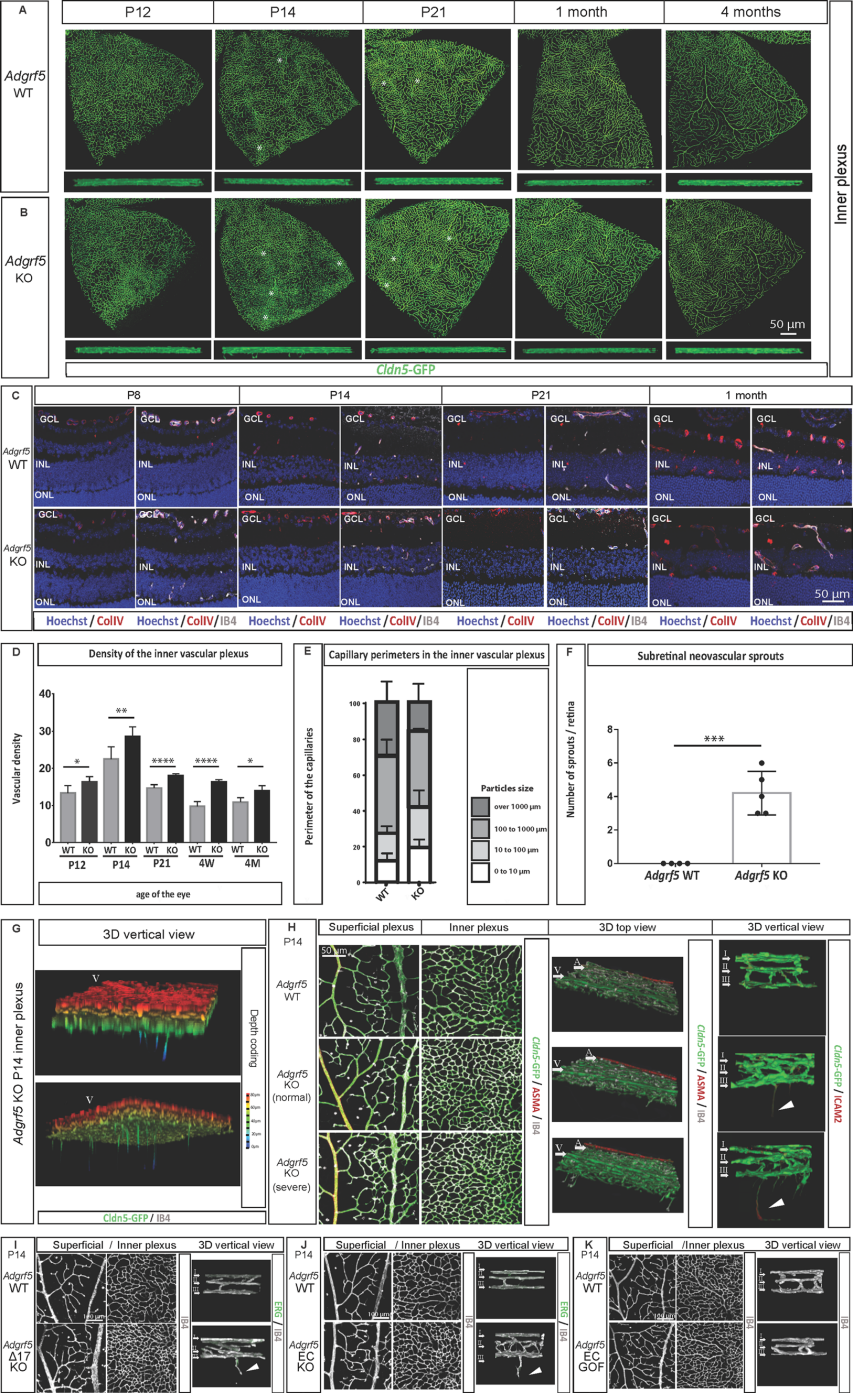
*Adgrf5* KO retina develops transient neovessels in the inner retinal space. **a** Inner vascular layer in a leaf of WT mouse retina at P12, P14, P21, 1 month, and 4 months. The endothelium is visualized in green tracking *Claudin5*-GFP. Horizontal view (upper panel) and optical cross-section (lower panel) are presented. Asterisks indicate areas of high vascular densities. **b** Inner vascular layer in a leaf of *Adgrf5* KO mouse retina at P12, P14, P21, 1 month, and 4 months. The endothelium is visualized in green tracking *Claudin5*-GFP. Horizontal view (upper panel) and optical cross-section (lower panel) are presented. **c** Images from *Adgrf5* WT (top row) and *Adgrf5* KO (lower row) mouse retina at P8, P14, P21, and 1 month. Cross-sectioned retinae were stained with collagen IV (red) and Hoechst (blue). The vasculature is depicted by IB4 staining (gray). **d** Quantification of the vascular densities in the inner plexus from *Adgrf5* WT or KO mouse retina at P12, P14, P21, and 1 month. **e** Quantification of the capillary perimeters in the inner plexus from *Adgrf5* WT or KO mouse retina at P14. **f** Quantification of the subretinal neovascular sprouts emerging from the inner plexus in *Adgrf5* WT or KO whole mouse retina at P14. **g** 3D reconstruction images of the three vascular layers below the vein of *Adgrf5* KO retina at P14. The retinal structure is presented in depth coding, displaying EC extensions after the inner vascular layer in the subretinal space (deep sprout is marked by an arrowhead). **h** Superficial (left) and inner (middle left) retinal vascular layer in-between an artery and a vein in WT and *Adgrf5* KO (normal and severe) mice at P14. The 3D reconstruction images of the three vascular layers and the neovessels below (middle right) are shown. IB4 (gray) and *Cldn5*-GFP (green) were used to label the endothelium, followed by ASMA staining to distinguish the artery and the vein. Vertical 3D projection of the three vascular layers and the neovessels below in the *Adgrf5* KO at high magnification (×63) (right) (deep sprout is marked by an arrowhead). **i** Superficial (left), inner (middle) vascular plexus and 3D view (right) from WT and Adgrf5 Δ17 KO at P14. The vascular layers were stained with IB4 (gray) (deep sprout is marked by an arrowhead). **j** Superficial (left), inner (middle) vascular plexus and 3D view (right) from WT and *Adgrf5* ECKO at P14. The vascular layers were stained with IB4 (gray) (deep sprout is marked by an arrowhead). **k** Superficial (left), inner (middle) vascular plexus and 3D view (right) from WT and *Adgrf5* ECGOF at P14. The vascular layers were stained with IB4 (gray) (deep sprout is marked by an arrowhead)

In order to explore the impact of ADGRF5-deficiency on the inner plexus morphology, we performed a longitudinal study. In the *Cldn5*-GFP WT mouse retina, the inner plexus that is initially composed of equal-sized and dense capillaries at P12 undergoes a progressive remodeling, which leads to the appearance of higher caliber vessels at P14. The inner network remodeled around those bigger vessels, becoming sparse and regular in the juvenile mice at P21 and 1 month of age, and this feature was further preserved over adulthood (Fig. 5a, top row). Interestingly, during the whole process, the inner plexus of WT mice was devoid of any deep sprouts that would dive into the outer nuclear layer (Fig. 5a, lower row). The inner plexus of *Adgrf5* KO retina, on the other hand, showed patches of capillaries with increased vascular density at P12 and P14, which were progressively normalized over age (Fig. 5b, quantified in Fig. 5d, e). Indeed, *Adgrf5* KO inner retinal layer was virtually indistinguishable from WT control group at 3 months (Fig. 5b, top row).

Vascular growth and maturation is accompanied by basement membrane deposition. The first sprouts reaching the inner plexus deposited very low amount of collagen IV (P14, Fig. 5c). As the inner plexus develops, the intensity of collagen IV-positive staining increased (P21, Fig. 5c) until it became very high in the mature vascular network (1 month, Fig. 5c). This increase in collagen IV deposition was delayed until P21 in the *Adgrf5* KO retinae, despite the extensive vascular development of the inner plexus (IB4-positive), implying a delay in maturation both at the morphogenetic and the basement membrane levels (Fig. 5c).

We noticed that the clusters of capillaries with high density in the inner plexus in the *Adgrf5* KO mouse retina coincided with sprouts emanating from the deep plexus toward the subretinal space at P14 (Fig. 5b, lower row, quantified in Fig. 5f, g). This led us to check where these cells originated from. By analyzing the endothelial expression pattern of *Cldn5*-GFP and lB4-positive cells, we found that these inner sprouts arose from the pre-existing capillaries forming the deep plexus. Additionally, the perivenous coalescent superficial network, the corresponding denser capillaries of the inner plexus, and the region of origin of the inner sprouts showed a close spatial correlation (Fig. 5h). We found that the more developed sprouts in the inner retinal space were closely associated with vascular clusters of very high density in the superficial plexus (Fig. 5h, *Adgrf5* KO (severe)).

Since the *Adgrf5* KO mice were generated by a large excision of the gene [28], spanning exon 3 to exon 21, a potential off-target effect due to either a deleted intronic sequence or the C57/Bl6 background could occur. To exclude these possibilities, we generated another mouse model harboring a mutation in exon 17 from the *Adgrf5* gene. This exon encodes for the seven transmembrane domains. Specifically, germ line deletion was achieved by crossing a male carrying the floxed exon 17 of the *Adgrf5* gene to a *Tie2*-Cre-positive female, since the activation of Cre recombinase by the *Tie2* promoter occurs during oocytogenesis. Thus, the floxed gene will be removed in zygote, ultimately resulting in global deletion of the gene [41]. The efficiency of the LoxP/Cre recombination in offspring was examined by PCR to select mutants that have lost exon 17 (Supplementary Figure 1a). Interbreeding of the offspring produced mutants and heterozygotes, which showed a complete or a half silencing of *Adgrf5* expression, respectively (Supplementary Figure 1b). This suggested instability of the mRNA after exon 17 excision. Next, we verified that this strain, called *Adrgf5* Δ17, can recapitulate the lung defects previously described in the *Adgrf5* KO [29–31]. Indeed, the *Adrgf5* Δ17 lung showed increased foamy cells and alveolar enlargement at 3 weeks of age (Supplementary Figure 1c). Moreover, 2-month-old *Adrgf5* Δ17 mice failed to retain a 1 kDa Alexa Fluor 555-cadaverine tracer in the CNS blood vessels, leading to tracer accumulation in the brain parenchyma (Supplementary Figure 1d). Overall, *Adrgf5* Δ17 mice recapitulated the main phenotypical abnormalities previously demonstrated in *Adgrf5* KO.

At P14, *Adrgf5* Δ17 retina exhibited a marked sprouting from the deep plexus toward the subretinal space compared to WT littermates (Fig. 5i, P14 *Adgrf5* Δ17), indicating that this phenotype is neither caused by the genetic background nor a neighboring effect in the full gene deletion model. Additionally, we examined *Adgrf5* ECKO mouse retina at P14 and observed sprouts diving from the deep plexus, confirming that the subretinal neovascularization phenotype observed upon loss of *Adgrf5* was endothelial-driven (Fig. 5j, P14 *Adgrf5* ECKO). Conversely, *Adgrf5* ECGOF retina showed no signs of dense EC clusters in the inner plexus, nor any subretinal sprouting at P14 (Fig. 5k).

**Fig. 6:**
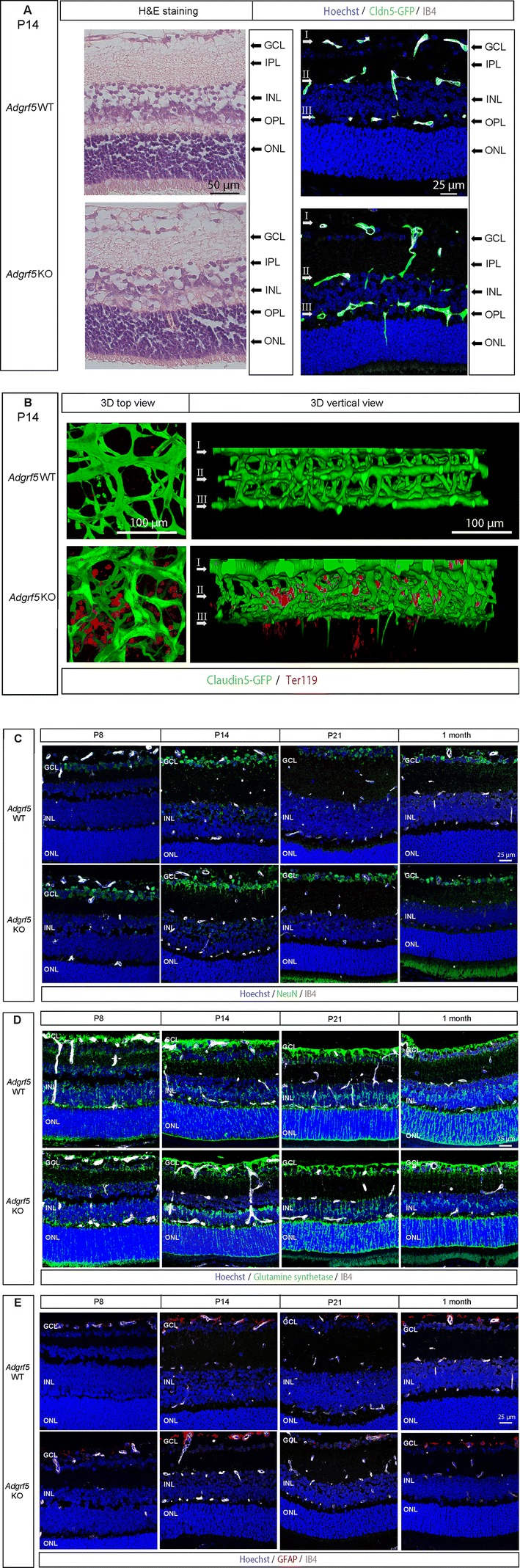
*Adgrf5* KO retinal tissue recovers after vessel normalization. **a** Images from WT and *Adgrf5* KO mouse retina at P14. Cross-sectioned retinae were stained with H&E (top) and Hoechst (blue). The vasculature is depicted by IB4 staining (gray) and *Claudin5*-GFP expression (green). **b** 3D reconstruction images of the three vascular layers of *Adgrf5* KO retina at P14. The retinal structure is presented viewed from the superficial layer (3D top view, left), or from a vertical view (right). Erythrocytes are visualized by Ter119 staining. **c** Images from WT and *Adgrf5* KO mouse retina at P8, P14, P21, and 1 month. Cross-sectioned retinae were stained with anti-NeuN antibody (green), Hoechst (blue), and IB4 (gray). **d** Images from WT and *Adgrf5* KO mouse retina at P8, P14, P21, and 1 month. Cross-sectioned retinae were stained with anti-Glutamine synthetase antibody (green), Hoechst (blue), and IB4 (gray). **e** Images from WT and *Adgrf5* KO mouse retina at P8, P14, P21, and 1 month. Cross-sectioned retinae were stained with anti-GFAP antibody (red), Hoechst (blue), and IB4 (gray)

Further histological characterization of the subretinal sprouts in *Adgrf5* KO using cross-sectioned retina revealed the presence of lesions descending from the OPL inside the ONL. The lesions were occasionally surrounded by erythrocytes, implying a possible defect in blood–retinal barrier establishment (Fig. 6a, H&E staining and Fig. 6b). Tracking double *Cldn5*-GFP- and IB4-positive labeling in retinal sections of the *Adgrf5* KO mice confirmed that neovessels penetrated the ONL, extending approximately 50 μm into the subretinal space, but seldom reached the outer segment (Fig. 6a, right column). These vascular lesions are reminiscent both in their sequence and histological nature of the early steps of RAP, where EC proliferation in the inner plexus precedes sprouts toward the subretinal space. They were not detected after P21 (Fig. 6c), and did not seem to anastomose with the choroidal vascular system (Supplementary Figure 2).

We investigated whether *Adgrf5* deficiency would result in retinal tissue damages around the vascular lesions. Besides the subretinal hemorrhages around the neovessels in the ONL (Fig. 6a), *Adgrf5* KO mouse retina displayed a disorganized GCL at P14 (NeuN staining, Fig. 6c). In agreement with the transient nature of the vascular lesions, the ganglion cells at later stages seemed intact. Glutamine synthetase staining results showed a normal development of Müller cells, which extended progressively from the GCL toward the inner plexiform layer, without any changes in the areas of subretinal neovascularization (Fig. 6d). GFAP staining, usually upregulated in situations of retinal stress, was confined to the GCL layer, confirming that the retinal damages were relatively limited (Fig. 6e). These results indicate that retinal gliosis did not occur in *Adgrf5* KO.

Overall, in line with the vascular normalization of the inner plexus over time in *Adgrf5* KO retinae, ADGRF5 deficiency does not cause any permanent histological abnormalities in adult mouse retina, suggesting a progressive recovery.

## Discussion

### *Adgrf5* deficiency results in abnormal retina patterning

This study extends our understanding of ADGRF5 function in patterning of the retinal vasculature. *Adgrf5* is strongly expressed in EC of different vascular beds, especially in the CNS endothelium. In fact, in the retina, only vascular endothelium seems to express *Adgrf5*. Yet *Adgrf5* loss-of-function was never linked to any obvious defect in the vascular architecture of any organ [28]. Although a severe impairment in the aortic arch arteries and the cardiac outflow tract formation was reported using a double *Adgrf5*/*Adgrl4* knockout mice, the phenotype did not seem to stem from an EC autonomous effect [38]. Here, we have shown that constitutive and endothelial-specific genetic ablation of *Adgrf5* leads to a transiently increased perivenous vascular density in the developing postnatal retina. These findings support for the first time the connection between an endothelial-specific loss of *Adgrf5* and a morphogenetic disorder that affects the vasculature.

The perivenous area is the latest region inside the superficial plexus undergoing vascular remodeling. Vascular remodeling encompasses multiple cellular events, such as cessation of proliferation, vascular pruning, and cell death, enabling nascent vessels to become fully functional. These events reshape the initial dense plexus into a sparser network, mostly from P10 to P14, starting in the vicinity of the artery and extending progressively toward the veins. Oxygen delivery, blood flow, and local cues are likely to mediate these events, although the balance between each of them remains poorly defined, especially along the artery-to-vein axis [42]. The cause of the incomplete vascular remodeling around the veins in *Adgrf5* KO retinae requires further exploration to determine whether this is due to altered blood flow, a pressure-related systemic dysregulation or an impaired local mechanism. The former hypothesis is supported by the predominant expression of *Adgrf5* on the arteriole side, whereas the defect seems to localize in the perivenous plexus (Figs. 3, 4). An impaired local mechanism would be in line with increasing evidence that aGPCRs can act as mechanosensors [43, 44].

### Perivenous endothelial cells link the superficial and the inner layer in developing retinal vasculature

We interrogated the role of the perivenous area during deep plexus formation. We have found that superficial and inner plexus are physically connected by forming vascular bridges in-between the two plexi at P9, as evidenced by using the endothelial *Cldn5*-GFP reporter mouse model. We also showed that vascular sprouts radiating from the perivenous region of the superficial plexus initiate the inner plexus formation (Figs. 2a–c). This process requires active EC migration, as demonstrated by genetic deletion of Rac1, a small Rho GTPase essential for cell migration in vivo [40]. Consequently, the absence of Rac1 significantly impinged on the progression of the perivenous sprouts toward the inner plexus (Fig. 2d). Our results suggest that Rac1 is not critical for *Adgrf5* retinal phenotype. Although ADGRF5 has been suggested to be coupled to RhoA and Rac1 in vitro [45], *Rac1* ECKO retinae revealed normal perivenous densities but severely reduced projections to the inner plexus, which was clearly different from the phenotype in *Adgrf5* KO mouse retinae displayed in Fig. 2d and [46].

To decipher the role of the superficial vascular plexus during the initial steps of inner plexus development, we have used *Adgrf5*-deficient mice as a genetic model to alter perivenous vascular density in the developing retina. As a consequence of the high perivenous vascular density in *Adgrf5* KO retinae, we observed an increased projection of vascular sprouts from these vessels toward the INL. This resulted in accelerated formation of the inner vascular plexus (P9) with very dense capillary patches below the vein (P14). These results suggest that the capillaries descending from the perivenous area are the main contributors to the inner plexus. Thus, an increased density in the superficial plexus in the perivenous area would consequently lead to inner plexus abnormalities. Yet, the pathways that control sprouts diving remain to be explored as most vascular guidance cues discovered to this point can modulate the bifurcation leading to new plexus formation [4, 5]. In particular, perivenous-derived sprouts could be endowed with a specific programing to dive into the retinal space, or alternatively the sub-venous area might be delineated by specific guidance cues.

### Superficial perivenous abnormalities can lead to transient vascular projections into the neuroretina

We observed that the abnormal clusters of capillaries observed in the inner plexus of *Adgrf5* KO retinae evolved into vascular projections inside the inner retinal space. RAP is a sub-class of pathological retinal neovascularization syndrome, characterized by neovessel growth from the inner plexus into the neuroretina [9]. Subretinal vascularization is usually defined as the second step toward a full RAP syndrome [10]. In most murine models of subretinal neovascularization, endothelial projections are detected around 2 weeks after birth. This coincides with massive developmental vascular remodeling of the inner vascular plexus, which at that time consists of very dense and rather randomly organized capillary networks. Our data suggest that this immature stage of the inner plexus development is strongly influenced by the overarching superficial plexus.

The mouse models currently available to examine subretinal vascularization are based on mutations that affect cell types distinct from the endothelium and disrupt the architecture of the neuroretina [47, 48], or that render the photoreceptor layer metabolically deprived, as established in the well-studied *Vldr* KO mouse model [15]. These findings demonstrated that alterations of the neuroretina, triggering cell-to-cell communication with the endothelium could eventually cause a burst of growth factors which influences the vascular invasion of the inner retina. Furthermore, the status of the endothelium using these mouse models has not been characterized in depth.

### Vascular invasion of the neuroretina as a function of the endothelium status

Intrinsic properties of the retinal endothelium might also have an impact on the RAP process. The first evidence for an important role of the endothelium was brought by the longitudinal study of mice carrying a vascular-specific, dominant-negative mutation in collagen type IV, alpha 1 (COL4A1) [16]. *Col4a1* mutant retina developed vascular lesions arising from the deep vascular plexus and extending toward the RPE where they formed chorioretinal anastomoses. Our study supports the role of the retinal endothelium in establishing subretinal neovascularization. As shown in Fig. 3, *Adgrf5* is exclusively expressed in the endothelium of the postnatal retinal vasculature. The *Adgrf5* KO mouse model, therefore, can be useful as a new model to study endothelial-driven subretinal vascularization.

The exact origin of subretinal neovascularization has been debated after the formal identification of retinal angiomatous proliferation as a subset of lesions distinct from AMD [10, 49]. Together with *Col4a1* mutants, our results from global and endothelial *Adgrf5* KO mice reinforce the possibility that RAP lesions directly originate from disturbed retinal vascular beds, without starting at the choroidal level.

Our data demonstrate that the vascular maturation process can considerably impact the subretinal vascularization. We have shown that upon remodeling, the superficial plexus is highly permissive for projections toward the inner plexus. Moreover, our results suggest that the maturation process of the inner plexus might exert a second level of control over the subretinal neovascularization: from P14 to P21, the inner plexus remodeling occurs and higher caliber vessels develop surrounded by a sparse network [4]. Indeed, this strong remodeling of the inner plexus appeared to abolish the deep sprouts in *Adgrf5* KO, and might represent a safeguard against subretinal neovascularization. In this regard, the expression of collagen IV, which was delayed in the inner plexus of *Adgrf5* KO, became normalized around that time point. Given the severe abnormalities seen in mouse retinae carrying a dominant-negative *Col4a1* mutation [16], it is possible that the basement membrane contains key maturation factors. The absence of any apparent histological alterations in adult *Adgrf5* KO retina also reinforces the idea of a protective normalization process.

Lastly, RAP is an age-related syndrome, and is assumed to affect mostly fully mature and established retinal vasculature. In adult mice, pharmacological abrogation of the shield offered by photoreceptor-produced sFlt-1 resulted in fast development of RAP lesions [17]. This indicates that some pathways are permanently required to maintain the vascular privilege. On the other hand, in most spontaneous RAP mice models, the subretinal vascularization occurs in the vascular bed undergoing active remodeling at P14. It is unclear whether these transient pathways are involved and reactivated in late-onset pathologies. It would be interesting to investigate whether an equivalent “permissive stage” as the one observed in the immature inner plexus observed in mouse (at P14, for instance) exists in human.

In summary, we have shown that the endothelium status is important for triggering subretinal vascularization. We have illustrated how the degree of development and maturation of the retinal inner plexus controls the appearance of deep sprouts. Moreover, alteration of *Adgrf5*, an endothelial-specific gene in retina, plays a critical role in shaping the inner plexus by regulating a pool of perivenous EC in the superficial plexus. Activating ADGRF5 could therefore help preventing the development of RAP lesions.

## Electronic supplementary material

Below is the link to the electronic supplementary material.

Supplementary material 1—Supplementary Fig. 1 associated with Fig. 3. Generation of *Adgrf5* Δ17. **A** Schematic of the PCR strategy (upper) and example of the PCR products (lower). Genomic DNA was used for genotyping from floxed *Adgrf5* control, or *Adgrf5* Δ17 mouse, wild-type (1), heterozygote (2), and knockout (3). **B** mRNA expression level of *Adgrf5* exon 2 and 3 from lung and brain assessed by qRT-PCR in *Adgrf5* Δ17 mouse at P14 (n = 4 mice per genotype). **C** Bright field view of the lung from 3 weeks old *Adrgf5* Δ17 and WT littermate controls. The lung sections were stained with H&E. Arrowheads are pointing at foamy macrophages. **D** Whole brain images taken after 1 kDa Alexa 555-cadaverine perfusion (left) and associated quantification of extravasated cadaverine (right) in 2 months-old WT and *Adrgf5* Δ17 mice (n = 3 mice for each genotype) (TIFF 6342 kb)

Supplementary material 2—Supplementary Fig. 2 associated with Fig. 6. Generation of *Adgrf5* Δ17. Images from WT and *Adgrf5* Δ17 mouse retina at P12, P14, 3 weeks, 1 month and 4 months. Cross-sectioned retinae were stained with H&E (TIFF 10481 kb)
