# Supplementary figures and images for: Adgrf5 contributes to patterning of the endothelial deep layer in retina
Source: Angiogenesis. 2019 Jun 29;22(4):491–505. doi: 10.1007/s10456-019-09674-0 (PMC6863953; doi:10.1007/s10456-019-09674-0)

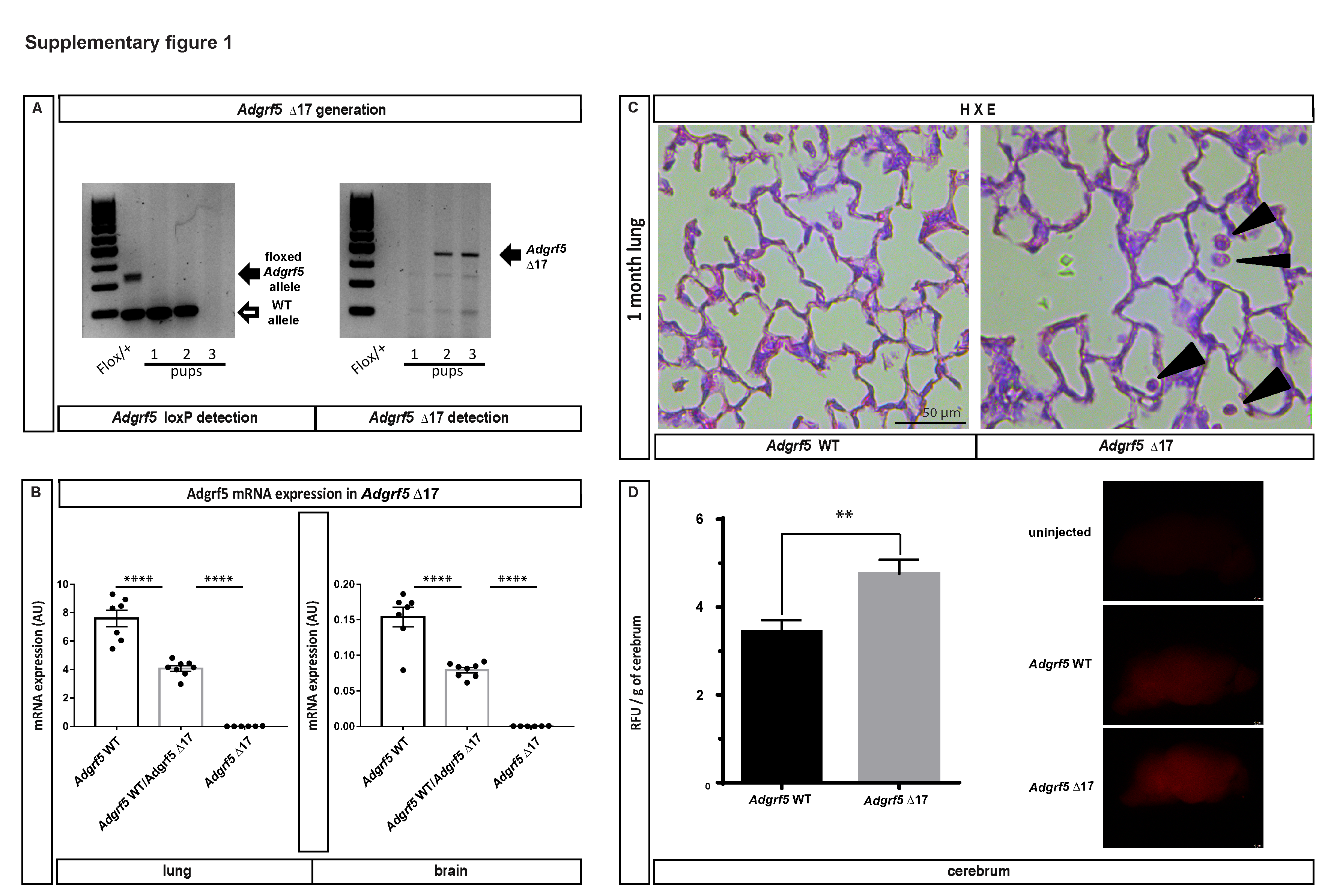

Supplement: Supplementary file 1 — Supplementary material 1—Supplementary Fig. 1 associated with Fig. 3. Generation of Adgrf5 Δ17. A Schematic of the PCR strategy (upper) and example of the PCR products (lower). Genomic DNA was used for genotyping from floxed Adgrf5 control, or Adgrf5 Δ17 mouse, wild-type (1), heterozygote (2), and knockout (3). B mRNA expression level of Adgrf5 exon 2 and 3 from lung and brain assessed by qRT-PCR in Adgrf5 Δ17 mouse at P14 (n = 4 mice per genotype). C Bright field view of the lung from 3 weeks old Adrgf5 Δ17 and WT littermate controls. The lung sections were stained with H&E. Arrowheads are pointing at foamy macrophages. D Whole brain images taken after 1 kDa Alexa 555-cadaverine perfusion (left) and associated quantification of extravasated cadaverine (right) in 2 months-old WT and Adrgf5 Δ17 mice (n = 3 mice for each genotype) (TIFF 6342 kb) [file 10456_2019_9674_MOESM1_ESM.tiff]

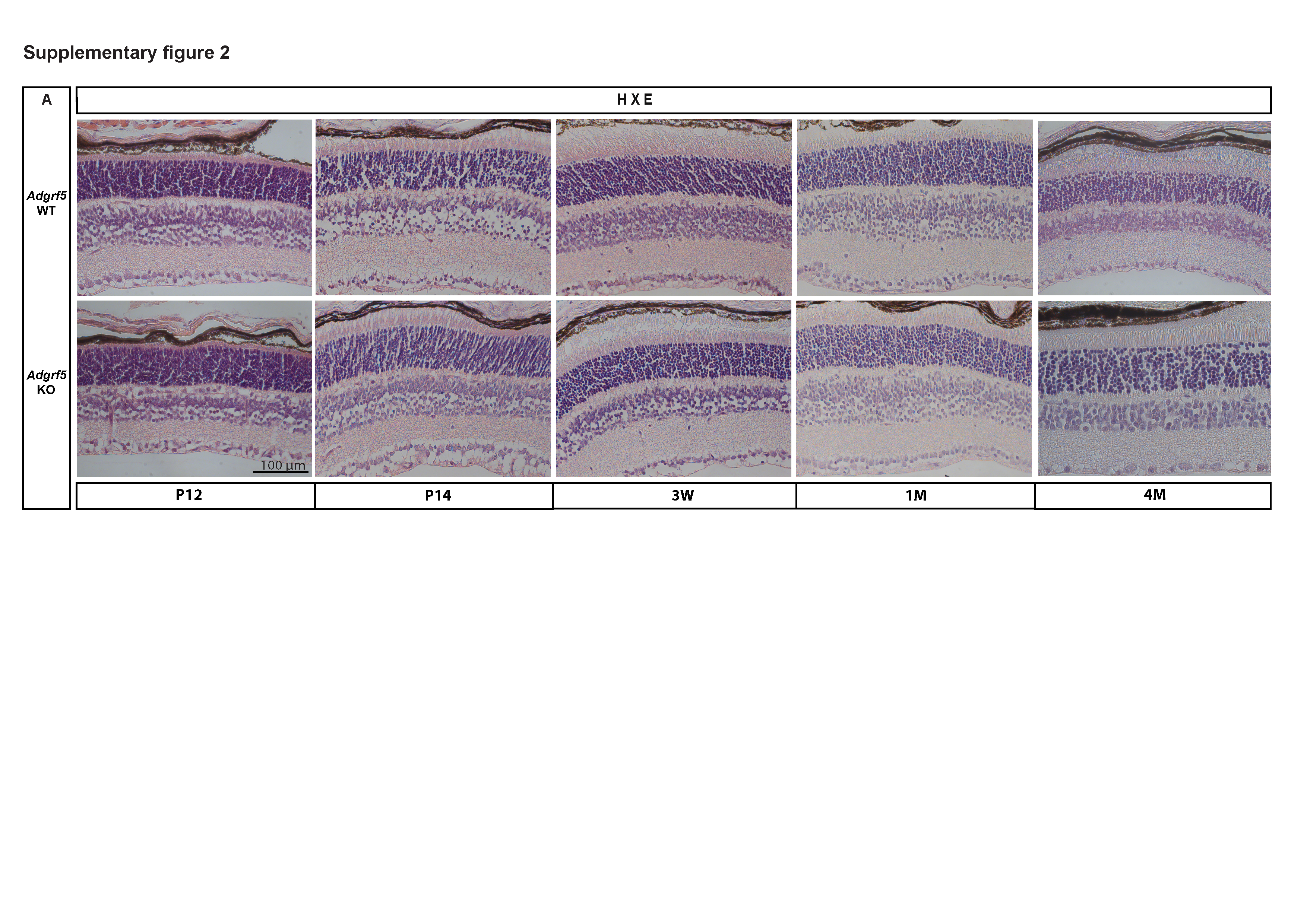

Supplement: Supplementary file 2 — Supplementary material 2—Supplementary Fig. 2 associated with Fig. 6. Generation of Adgrf5 Δ17. Images from WT and Adgrf5 Δ17 mouse retina at P12, P14, 3 weeks, 1 month and 4 months. Cross-sectioned retinae were stained with H&E (TIFF 10481 kb) [file 10456_2019_9674_MOESM2_ESM.tiff]
